# In-Silico Design, Synthesis and Evaluation of a Nanostructured Hydrogel as a Dimethoate Removal Agent

**DOI:** 10.3390/nano8010023

**Published:** 2018-01-04

**Authors:** Fabian Avila-Salas, Adolfo Marican, Jorge Villaseñor, Mauricio Arenas-Salinas, Yerko Argandoña, Julio Caballero, Esteban F. Durán-Lara

**Affiliations:** 1 Centro de Nanotecnología Aplicada, Facultad de Ciencias, Universidad Mayor, Huechuraba 8580000, Chile; fabian.avila@umayor.cl; 2 Centro de Bioinformática y Simulación Molecular, Facultad de Ingeniería, Universidad de Talca, Talca 3460000, Chile; marenas@utalca.cl (M.A.-S.); yargandona@utalca.cl (Y.A.); 3 Instituto de Química de Recursos Naturales, Universidad de Talca, Talca 3460000, Chile; amarican@utalca.cl (A.M.); jvillase@utalca.cl (J.V.); 4 Biomaterials and Drug Delivery Laboratory, Núcleo Científico Multidisciplinario, Dirección de Investigación, Universidad de Talca, Talca 3460000, Chile

**Keywords:** cross-linked PVA hydrogel, biodegradable, dimethoate, pesticide, absorption

## Abstract

This study describes the in-silico design, synthesis, and evaluation of a cross-linked PVA hydrogel (CLPH) for the absorption of organophosphorus pesticide dimethoate from aqueous solutions. The crosslinking effectiveness of 14 dicarboxilic acids was evaluated through in-silico studies using semiempirical quantum mechanical calculations. According to the theoretical studies, the nanopore of PVA cross-linked with malic acid (CLPH-MA) showed the best interaction energy with dimethoate. Later, using all-atom molecular dynamics simulations, three hydrogels with different proportions of PVA:MA (10:2, 10:4, and 10:6) were used to evaluate their interactions with dimethoate. These results showed that the suitable crosslinking degree for improving the affinity for the pesticide was with 20% (*W*%) of the cross-linker. In the experimental absorption study, the synthesized CLPH-MA20 recovered 100% of dimethoate from aqueous solutions. Therefore, the theoretical data were correlated with the experimental studies. Surface morphology of CLPH-MA20 by Scanning Electron Microscopy (SEM) was analyzed. In conclusion, the ability of CLPH-MA20 to remove dimethoate could be used as a technological alternative for the treatment of contaminated water.

## 1. Introduction

The treatment of water that is contaminated with organophosphorus (OP) compounds remains until today as a problem worldwide, which needs to be solved with novel and innocuous technologies. The OP compounds are organic molecules containing phosphate groups that have the capacity to irreversibly inactivate the enzyme acetylcholinesterase (AChE) [1]. Human exposure to OP compounds can decrease the activity of vital neurotransmitters, resulting in incapacitating symptoms that vary from rhinorrhea, excessive salivation, perspiration, lacrimation, headaches, nausea, vomiting, abdominal pain, chest tightness, dyspnea, involuntary urination and defecation, muscle fasciculation, seizures, coma, and potentially even death [2,3]. Dimethoate (*O*,*O*-dimethyl-*S*-methylcarbamoyl methylphosphorothioate), or DMT, is one of the most widely used OP compounds [4] (Table 1). This compound is widely used as an insecticide on crops (e.g., wheat, alfalfa, corn, and cotton), orchards, in forestry, and for residential purposes. DMT is of great concern because of its toxicity and potentially harmful effects on water sources [5]. Specifically, the extreme use of DMT could lead to excessive residues accumulating in the environment and in the human body through the food chain, which could cause death [6,7].

Several methods have been developed and implemented to remove OP compounds from contaminated water, such as solid phase extraction, photo-catalytic methods, advanced oxidation processes, ion exchange, and absorption processes [8,9]. However, these methods and processes have associated drawbacks such as: high operating costs, poor efficiency, the use of chemicals with high toxicity, and secondary generation of waste that is harmful to humans and the environment. In these circumstances, the absorption techniques using highly absorbent materials, such as polymer hydrogels, have received great attention owing to their easy availability, bio-compatible nature, and high efficiency for the removal of unwanted and dangerous molecules from aqueous solutions [10,11,12].

Hydrogels are three-dimensional hydrophilic polymer networks with a suitable crosslinking degree [13]. Their reticulated structures are able to generate surface pockets and micro/nano-pores that enhance their capacities to absorb large amounts of water or target molecules, but do not dissolve when brought into contact with water [14] According to the kind of crosslinking reaction, the hydrogels can be categorized into two groups: physical hydrogels and chemical hydrogels [15]. Physical hydrogels maintain their network structural integrity through non-covalent interactions, such as: hydrogen bonds (hbonds) [16], ionic/electrostatic interactions [17], and van der Waals (vdW) interactions [18]. Chemical hydrogels adopt their network structure through the formation of covalent bonds that are generated by the crosslinking agents [19,20].

**Figure 1:**
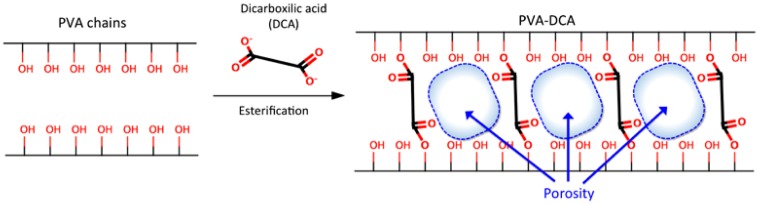
Schematic representation of the esterification process that allows for the formation of covalent bonds between Polyvinyl Alcohol (PVA) chains and dicarboxylic acids.

The highly porous structure of the hydrogels can be easily tuned by controlling the density of the crosslinking agents (crosslinking degree) [21]. Therefore, the structural properties of hydrogel and its affinity for certain bioactive molecules will depend directly on the selection of constituent polymers and the type of crosslinking agent that will form the polymer cross-linked mesh. When considering the above, polyvinyl alcohol (PVA) polymer was selected because it has been used with good results as the base material for the generation of hydrogels for multiple applications [22], in addition, it is a non-toxic and low-cost polymer [23]. The preparation of PVA hydrogels can be performed by chemical methods, which involve the formation of interactions and bonds between the PVA chains and the functional groups of the crosslinking agents [24]. The concentration of crosslinking agents affects the porous structure, swelling features, and mechanical strength of the hydrogel. The use of dicarboxilic acids as crosslinking agents generates flexible and transparent PVA hydrogels [24] that are capable of interacting with water-soluble compounds [25]. These acids have two carboxylic functional groups at both ends of their structure, which through an esterification process, can generate covalent bonds with the hydroxyl groups that are located in the PVA chains (Figure 1), generating the crosslinking and the hydrogel structural porosity [24].

The aim of the present study was to design, synthesize, and evaluate a non-toxic and low-cost hydrogel based on cross-linked poly (vinyl alcohol) for the absorption of DMT from aqueous solutions. The effectiveness as crosslinking agent of 14 dicarboxilic acids was evaluated using a semiempirical quantum mechanical methodology [4,12,26,27,28], which allowed for estimating the interaction energy of nano-pore/DMT complexes. The experimental absorption capacity of the superabsorbent hydrogel as a strategy to remove DMT from aqueous solution was determined. These results were correlated with the theoretical data obtained from molecular dynamics simulations (MDS) studies.

## 2. .Results and Discussion

### 2.1. In-Silico Interaction Energy 

Table 2 shows the values of interaction energies calculated between DMT and each PVA nano-pore (PVAnp) formed by the different dicarboxilic acids that were covalently bonded to short PVA chains. These calculations allowed for quickly evaluating the contribution of the structural features of each crosslinking agent in the obtained interaction energy values. The results showed that the PVAnp generated with malic acid (MA) had the best interaction energy, and therefore, MA was the candidate chosen to carry out the PVA crosslinking since it could generate nano-pores with the suitable size for enhancing their interaction with DMT.

### 2.2. Molecular Dynamics Simulations (MDS) Studies

MDS studies were performed in order to understand the molecular behavior and the interactions between DMT molecules and three cross-linked PVA hydrogels (CLPH), whose cross-linking process was carried out using three concentrations of MA: 20, 40, and 60-wt % (CLPH-MA20, CLPH-MA40, and CLPH-MA60 hydrogels, respectively). The hydrogels and DMT molecules were immersed in water boxes and MDS were carried out while considering 100 ns of simulation.

**Figure 2:**
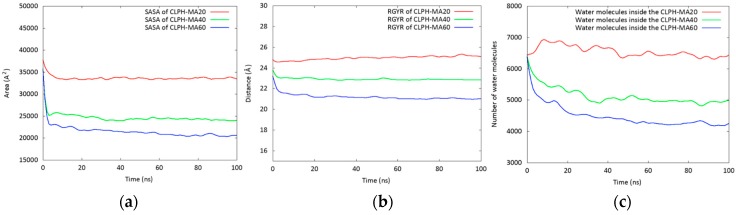
(**a**) Solvent Accessible Surface Area (SASA) and (**b**) Radius of Gyration (RGYR) plots of the three hydrogels; (**c**) Number of water molecules inside the hydrogels.

**Figure 3:**
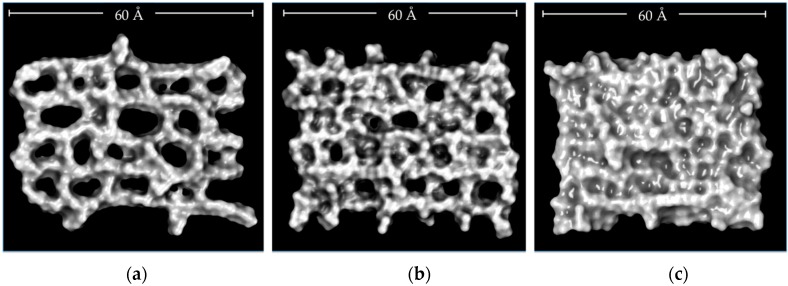
In-silico representation of the structural porosity generated during the MDS due to the crosslinking degrees of (**a**) CLPH-MA20; (**b**) CLPH-MA40, and (**c**) CLPH-MA60.

Figure 2a shows calculated Solvent Accessible Surface Area (SASA) values in order to quantify the accessible area of each hydrogel that can interact with both the solvent and the DMT molecules. CLPH-MA20 showed an approximate SASA of 3500 A^2^, which was 30% and 40% greater than CLPH-MA40 and CLPH-MA60, respectively. This difference was due to the crosslinking degrees of the hydrogels, where CLPH-MA40 and CLPH-MA60 had higher density of crosslinking agents bonded to the PVA chains. Therefore, they showed a more rigid and compact structure (Figure 3b,c), unlike CLPH-MA20, which showed a high structural porosity (Figure 3a). Similar trends were observed when calculating the Radius of Gyration (RGYR) of each hydrogel (Figure 2b). RGYR represents the mean distance between each atom and the center of mass of the hydrogel, and was a useful value to provide a structural measure of the degree of compaction of the studied hydrogels with MDS. Figure 2b depicts the evolution of the RGYR of each hydrogel over the full MDS trajectories. CLPH-MA20 displayed a greater RGYR over time, reaching near 25 Å. The RGYR of CLPH-MA40 (around 23 Å) and CLPH-MA60 (21 Å) remained remarkably stable over time, suggesting that both of the hydrogels reached a more compact final structure (Figure 3b,c), in comparison to CLPH-MA20 (Figure 3a).

The role of the hydration in the conformational structure of each hydrogel was determined by counting the number of water molecules within 3.0 Å of the hydrogel backbone and all the rest of the water molecules located at a maximum distance of 25 Å, 24 Å, and 21 Å from the center of mass of each hydrogel (CLPH-MA20, CLPH-MA40, and CLPH-MA60, respectively). These maximum distances correspond to the calculated RGYR. Figure 2c shows the behavior of the water molecules inside the hydrogels during the MDS trajectories. It is possible to observe that the porous and flexible structure of CLPH-MA20 allowed for the interaction and incorporation of around 6500 water molecules inside its structure, which was 25% and 35% greater than CLPH-MA40 and CLPH-MA60, respectively.

**Figure 4:**
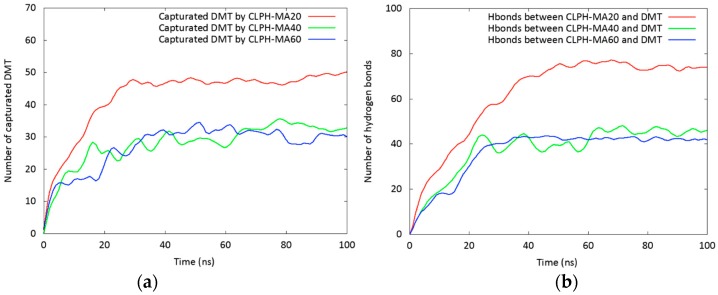
(**a**) Plot of DMT molecules captured by the three hydrogels; (**b**) number of hydrogen bonds identified during the simulation.

**Figure 5:**
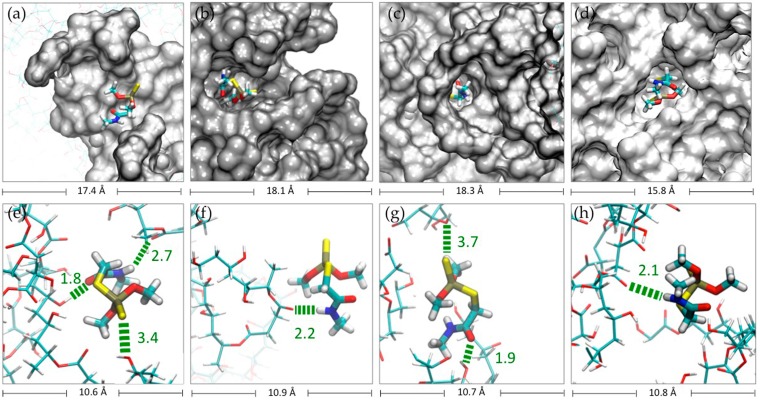
DMT interaction with (**a**,**b**) surface pocket and (**c**,**d**) nano-pores of CLPH-MA20. (**e**–**h**) Molecular Dynamic Simulation (MDS) snapshots of the possible hbond interactions between DMT molecules and CLPH-MA20.

The capture of DMT by each hydrogel during the 100 ns of simulation is shown in Figure 4a. It was considered a contact distance of 4.5 Å between the CLPH-MA backbone and DMT molecules. CLPH-MA20 captured 100% of DMT when reaching 40 ns, remaining stable during the rest of the simulation. CLPH-MA40 and CLPH-MA60 only were able to capture up to 65% and 55% of DMT, respectively (Figure 4a). The lower capture of these last two hydrogels was due to the fact that their more compact structures (Figure 3b,c) impeded an efficient interaction with the DMT molecules, which did not adhere to the nano-cavities that generated on the surface of the hydrogels. On the contrary, CLPH-MA20 has nano-pores (Figure 5c,d) that for allowed the absorption of DMT molecules inside its structure; in addition, it has superficial nano-pockets where DMT could be efficiently adhered (Figure 5a,b).

MDS studies allowed observing the binding interactions between PVA hydrogels and DMT molecules. Hydrogen bonds between each hydrogel and the DMT molecules were computed (Figure 4b). There was a direct relationship between the capture of DMT and the number of hydrogen bonds that are generated with each hydrogel. At 40 ns of simulation (Figure 4b), CLPH-MA20 maintained stable more than 70 hbonds, which would indicate that a considerable number of DMT molecules interacted with the hydrogel through two or three hbonds simultaneously (Figure 5e,g).

DMT has in its structure different functional groups that are capable of generating different hbonds: an amide group that owns a carbonyl (C=O) where the oxygen acts as hbond acceptor, a secondary amine (N–H), which allows amide to function as a hbond donor as well, and a sulfur group, which is able to act as a hbond acceptor. Thus, DMT was able to establish up to three hbonds with PVA hydrogel (Figure 5e), where the carbonyl oxygen and the sulfur could form hbonds with the hydroxyl groups of PVA chains and hydroxyl groups of the crosslinked malic acids (Figure 5e,g). Moreover, the N–H group could form hbonds with carbonyl oxygen of the crosslinked malic acids (Figure 5e,g,h).

### 2.3. Characterization of CLPH-MA20 by Scanning Electron Microcopy (SEM)

**Figure 6:**
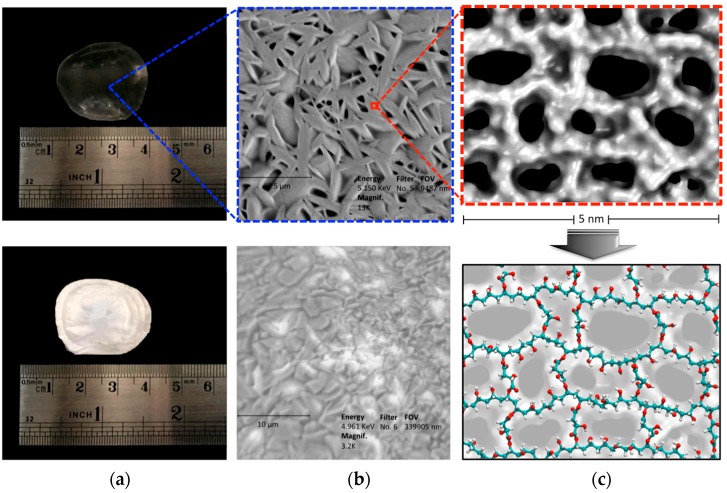
(**a**) Photographs of a small disk of CLPH-MA20, without DMT (the top image) and with DMT (the bottom image). (**b**) SEM micrographs of CLPH-MA20 without DMT (the top image) and with DMT (the bottom image). All hydrogels were lyophilized after the swelling process. (**c**) Snapshots taken from MDS studies, where the top image shows the possible conformational structures of the nano-pores, the bottom image shows the PVA chains cross-linked with malic acid that generated nano-porosity.

Figure 6 shows photographs and SEM micrographs of the dried samples of the CLPH-MA20. The top image (Figure 6a) depicts the CLPH-MA20 without DMT; the presentation of this formulation was transparent unlike the whitish appearance of CLPH-MA20 with trapped DMT showed in the bottom image of Figure 6a. The SEM technique allowed for obtaining differences in morphology in both stages of the hydrogel, the top image in Figure 6b shows CLPH-MA20 without DMT, which has the smooth walls, an assembly of marked fiber networks, and the high micro-porous structure with well-defined shapes that exhibit some spread in pore size, this type of porosity structure could play a key role in enhancing the DMT diffusion throughout the hydrogel. The bottom image of Figure 6b shows CLPH-MA20 swollen in DMT with concentrations of 500 mg L^−1^. Figure 6c shows snapshots taken from MDS studies, the top image shows the possible conformational structures of the nano-pores, and the bottom image shows the PVA chains cross-linked with malic acid that generated this nano-porosity.

### 2.4. Absorption Kinetics of DMT

Table 3 shows the values obtained in the experimental design. It is observed that the retention percentage of DMT reached 89.28% at the lowest interaction time (10 min) and at the lowest hydrogel mass (34.2 mg). Subsequently, the hydrogel was capable of retaining all of the DMT in the solution at any hydrogel mass and time value, in the intervals studied.

**Figure 7:**
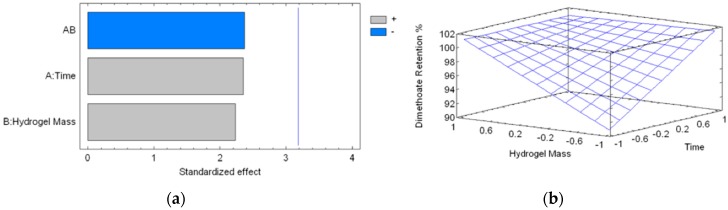
(**a**) Standardized Pareto chart for DMT retention percentage due to hydrogel treatment (Where: A is the time of reaction, B is the hydrogel mass and AB is the interaction). The line represents the critical *t*-value, 95% confidence; and, (**b**) estimated response surface.

The interaction between the hydrogel and DMT in aqueous solution (at pH 5.5) is presented in Figure 7. The Pareto chart (Figure 7a) shows that none of the variables were statistically significant. In spite of this, time and hydrogel mass exerted a positive influence; meanwhile, the interaction of these variables exerted a negative influence on the DMT retention percentage. The estimated response surface (Figure 7b) showed that the percentage of retention increased when the time of contact and hydrogel mass were increased, reaching a maximum value at the end of each interval.

The regression Equation (1) of the model is:

DMT Retention% = 98.6 + 2.66 × *A* + 2.53 × *B* − 2.70 × *AB* (R^2^ = 84.35) (1)

The optimum experimental conditions for the capture of this compound were as follows: an interaction time of 90 min, and a hydrogel dose of 10 mg·mL^−1^. Although none of the variables were statistically significant, it is preferred to give adequate contact time so that the retention of DMT is optimal.

## 3. Materials and Methods

### 3.1. Theoretical Section

#### 3.1.1. Building Molecular Structures

Structures of DMT, 14 dicarboxilic acids, PVA monomer, and PVA nano-pores (PVAnp) were built using GaussView program version 3.09 (Semichem, Inc., Shawnee Mission, KS, USA) [29], when considering their protonation states at pH 5.5. Their three-dimensional structures were optimized using the software Gaussian version 03 Inc. (Wallingford, CT, USA) [30] at Density Functional Theory (DFT) level with B3LYP method and 6–311G+(d,p) basis set. These dicarboxilic acids (Table 4) were selected from different studies, in which the formation of hydrogels was successfully carried out [31,32,33,34,35,36,37,38,39].

#### 3.1.2. In-Silico Calculation of Interaction Energy

**Figure 8:**
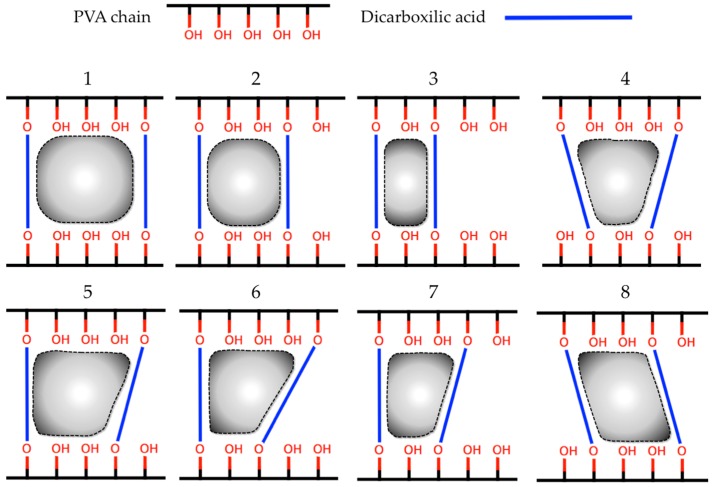
Different forms of nano-pores generated between short chains of PVA covalently bonded with pairs of dicarboxylic acids.

A semi-empirical quantum mechanical strategy, complemented with Monte Carlo conformational sampling [26,27,28], was used to calculate the interaction energy of molecule1–molecule2 complexes. In this case, molecule1 represents each one of the 14 PVAnp (Figure 8) and molecule2 represents DMT. For the construction of each PVAnp, two PVA chains (of five monomers, equal to 10 Å, which represent the maximum length of DMT) were covalently bonded by pairs of dicarboxylic acids, as indicated in Figure 8.

To calculate the interaction energies, a semi-empirical approach was employed, which is briefly described below: (i) the mass centers of molecule1 and molecule2 were placed near of the origin of the cartesian coordinates frame; (ii) the molcule1 was selected to remain static, then the new orientation of molecule2 (in relation to molecule1) was chosen according to a set of three Euler angles (α, β, γ) randomly obtained [40]; (iii) molecule2 was then translated along the random vector [40] until the vdW surfaces of each molecule touch each other; (iv) after translation, single-point energy (1SCF) for this specific molecular conformation (molecule1–molecule2 complex) was calculated using Parameterized Model number 7 (PM7) [41], which is a semi-empirical quantum mechanical method that is implemented in MOPAC2016 software version 16.111L (for LINUX, Colorado Springs, CO, USA) [42]; (v) the total energy (*E*) was extracted of the previous 1SCF calculation and also from their isolated parts. Then, a super molecular approach was used to obtain directly the interaction energy (Δ*E*) as the difference between the energy of molecule1–molecule2 complex and the sum of the energies of its isolated parts. This was defined with the following Equation (2):

Δ*E*_1,2_ = *E*_(molecule1–molecule2)_ − (*E*_(molecule1)_ + *E*_(molecule2)_) (2)

Thus, steps (i) to (v) were repeated up to generate of 100 thousand different molecular configurations and their corresponding interaction energies. Finally, the average of the interaction energies calculated for the eight nano-pores for each type of dicarboxylic acid was obtained.

#### 3.1.3. Molecular Dynamic Simulation (MDS)

LEAP module of AmberTools17 software version 17.05 (for LINUX, University of California, San Francisco, CA, USA) [43] was used to generate 25 PVA chains of 25 monomers long each one. Subsequently, using PACKMOL software version 16.070.3 (for LINUX) [44] these chains were randomly distributed within a three-dimensional box of 70 Å × 70 Å × 70 Å (*X*, *Y*, and *Z* axes). The chains were separated one from each other by a distance of at least 5 Å. The LEAP module was used to perform the crosslinking procedure (Figure S1), which is based on a cyclic iteration scheme, each cycle consists of three steps: (1) random selection of two OH groups of two different PVA chains (separated by no more than 10 Å), (2) covalent bonding of these –OH groups with the –COO^−^ groups of MA), the modified polymer system is saved in a MOL2 file, whose format is corrected assigning to each atom a different identifier using Antechamber software package version 1.27 (for LINUX) [45], (3) minimization of the modified polymer system (in order to avoid steric hindrance), using the steepest descent algorithm and the Universal Force Field (UFF) implemented in Openbabel software version 2.3.1 (for LINUX) [46]. When considering that the polymer matrix had a total of 625 PVA monomers, the steps from 1 to 3 were repeated until to incorporate 125, 250, and 375 MA inside each polymer matrix. In this way, according to the established proportions of PVA monomers:MA 10:2, 10:4, and 10:6, three cross-linked PVA matrices were obtained: CLPH-MA20, CLPH-MA40, and CLPH-MA60.

PACKMOL software version 16.070.3 (for LINUX) [44] was used to randomly add 50 DMT molecules around each cross-linked PVA matrix (when considering a separation distance of 10 Å). Using the “System Builder Module” of Desmond/Maestro software academic version 4.4 (for LINUX) [47], the final systems were added in the center of solvent boxes of the following size: 110 Å, 110 Å, 110 Å (axes *X*, *Y*, *Z*, respectively). Subsequently, the boxes were solvated with TIP3 water molecules. The default relaxation protocol implemented in Desmond/Maestro software version 4.4 (for LINUX, DE Shaw Research, New York, NY, USA) [47] was used. Briefly, this protocol consisted in a series of steps in which the molecular systems were firstly energy minimized using a steepest descent algorithm switching on and switching off restraints over heavy atoms. Then, a series of four short NVT (constant number (N), volume (V), and temperature (T)) and NPT (constant number (N), pressure (P), and temperature (T)) MDS (of 12 and 24 ps) were performed retaining restraints to finally perform an unrestrained simulation. The parameters of the final MDS were as follows: a NPT ensemble was used keeping the temperature at 300 K by means of the Nosé–Hoover chain method, with a relaxation time of 1.0 ps. Pressure was kept fixed at 1.0 bar using the Martyna–Tobias–Klein (MTK) barostat using an isotropic coupling and a relaxation time of 2.0 ps. The RESPA integrator was used to integrate the equations of motions with a 2.0 fs time step for bonded and near interactions and a 6.0 fs time step for far interactions. A cutoff radius of 9 Å was used for non-bonding interactions (van der Waals and electrostatic interactions). The OPLS force field was applied automatically to assign the standard charges and parameters to the systems. Finally, three MDS were run using about 100 ns.

From the results of the MDS, 1000 frames were extracted for analysis. The Solvent Accessible Surface Area (SASA) [48], the Radius of Gyration (RGYR) [49,50], and the capture of DMT (within a distance of 4.5 Å with respect to any atom of the CLPH) were calculated with TCL scripts using VMD software version 1.9.2 (for LINUX) [51]. These were comparatively plotted using the Gnuplot software version 5.0 [52]. Employing the Discovery Studio Visualizer tools (DS Visualizer) version 4.1. Accelrys Software Inc., San Diego, CA, USA [53], the intermolecular interactions between CLPHs and DMT were analyzed.

### 3.2. Experimental Section

#### 3.2.1. Materials

Polyvinyl Alcohol (PVA) 30–60 KDa, Malic Acid, NaHCO_3_, acetonitrile (HPLC grade) and MMP analytical standards were purchased from Sigma (St. Louis, MO, USA), HCl and Methanol (HPLC grade) were purchased from Merck (Darmstadt, Germany). All of the solutions were prepared using MilliQ water. 

#### 3.2.2. Synthesis and Characterization of CLPH-MA

Three CLPH-MA hydrogels with different crosslinking degrees (10:2, 10:4, and 10:6) were synthetized and characterized by Fourier-Transform Infrared Spectroscopy (FT-IR) (Nicolet Nexus 470 FT-IR, Thermo Scientific, Waltham, MA, USA) and Thermogravimetric analysis (TGA Q500) (TA Instruments, New Castle, DE, USA), as previously reported [12]. As a brief description, the synthesis of a series of CLPH-MA was carried out through the esterification of PVA with MA. The final concentrations of MA were 20, 40, and 60-wt % for CLPH-MA20, CLPH-MA40, and CLPH-MA60 hydrogels, respectively. Moreover, the swelling and degradation studies were performed as previously reported [12].

#### 3.2.3. SEM Analysis

The sample was cut and loaded in the copper stub. Later, it was stained with 0.7% (*w*/*v*) phosphotungstic acid, washed, and air-dried. The sample was examined in SEM mode, in a low-voltage electron microscope (LVEM) (Delong Instruments s.r.o., Brno, Czech Republic), and was used at a nominal operating voltage of 5 kV (LVEM5).

#### 3.2.4. Absorption Kinetic of DMT by CLPH-MA20 in Model Solutions

The absorption kinetic study of DMT by CLPH-MA20 was evaluated by previously determining the percentage decrease in the absorbance at each specific maximum absorbance wavelength, using the following Equation (3):

Absorption% = (*A*_0_ − *A*)/*A*_0_ × 100 (3)

where *A*_0_ is the initial absorbance at a specific wavelength and *A* is the final absorbance at the same wavelength (Table 1). A model solution of 500 mg·L^−1^ DMT in Milli-Q water was used for all of the assays of absorption by CLPH-MA. Between 30 and 100 mg of CLPH-MA20 were used per test, using 10 mL of model solution in each experiment. The times selected in this study were obtained from the statistical design, as discussed in Section 3.2.5. The Absorption Kinetic model was performed in distillated water (pH 5.5) at room temperature.

The pKa of carboxylic acids are around 4.7 whereas of hydroxyl groups are around 10.6, as reported in the literature [54,55]. The pH of experimental testing was performed in the distilled water at pH 5.5. Therefore, it considers that at this pH, the carboxylic acids are deprotonated, as depicted in Table 4. On the other hand, hydroxyl groups from PVA are protonated at pH 5.5 and only are deprotonated when they interact with the linkers during the esterification process, with the aim of generating covalent bonds.

#### 3.2.5. Statistical Analysis

3 mL of an aqueous solution of DMT of 500 ppm was used, and the percentage of retention by the hydrogel was evaluated. An experimental design of 2^2^ + 3 center points was used, when considering a contact time between 10 and 90 min, and a hydrogel mass between 34.2 and 101.3 mg (Table 3). With this experimental design, it was possible to determine the best experimental conditions for retention of DMT by CLPH-MA20. The values of the experimental variables were coded between −1 and 1 to have the same statistical weight.

## 4. Conclusions

An in-silico methodology has been implemented to rapidly evaluate the affinity of DMT with hydrogels that were crosslinked with different crosslinking agents based on dicarboxilic acids. To do this, nano-pores of different sizes and shapes were designed, for which their interaction energies with DMT were calculated using semiempirical methods of quantum mechanics. The results showed that malic acid (MA) was the best candidate to carry out the cross-linking of the PVA hydrogel. For this reason, we have designed and synthesized a super adsorbent material that is based on a hydrogel of PVA crosslinked with MA for the removal of DMT from aqueous solutions.

The absorption process of pesticides seems to be controlled by several factors, including the crosslinking degree, number and size of pores, time of contact, and types of intermolecular interactions that can be formed with the hydrogel. MDS studies allowed for elucidating that the high affinity was due to multiple hydrogen bonds that occurred between the secondary amine, carbonyl, and sulfur groups from DMT and the hydroxyl and carbonyl groups available mainly in the PVA-MA chains. According to our studies, this super absorbent material based on crosslinked PVA hydrogel using MA as a crosslinker had an excellent absorption efficiency for the removal of DMT; the CLPH-MA20 was able to trap the 100% of the pesticide studied.

## Figures and Tables

**Table 1:** Structure and properties of Dimethoate (*O*,*O*-dimethyl-*S*-methylcarbamoyl methylphosphorothioate) (DMT).

Chemical structure	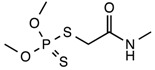
Molecular formula	C_5_H_12_NO_3_PS_2_
Appearance	White crystalline solid
Solubility in water (20–25 °C)	More than 5000 mg/L water
Mol. Wt.	229.26 g·mol^−1^
Melting point	51–52 °C
Wavelength (λ, nm)	280 nm

**Table 2:** Average interaction energy values calculated using semi-empirical of quantum mechanics methods.

Id.	Hydrogel	Average Interaction Energy Kcal/mol	Id.	Hydrogel	Average Interaction Energy Kcal/mol
1	PVAnp-Oxalic acid	−1.861	8	PVAnp-Itaconic acid	−1.988
2	PVAnp-Malonic acid	−1.952	9	PVAnp-Tartaric acid	−1.879
3	PVAnp-Succinic acid	−1.955	10	PVAnp-Glutaric acid	−1.961
4	PVAnp-Malic acid	−1.998	11	PVAnp-Adipic acid	−1.985
5	PVAnp-Fumaric acid	−1.994	12	PVAnp-Pimelic acid	−1.975
6	PVAnp-Maleic acid	−1.964	13	PVAnp-Suberic acid	−1.969
7	PVAnp-Citraconic acid	−1.993	14	PVAnp-Azelaic acid	−1.924

**Table 3:** Experiments performed to determine the percentage of retention of DMT by the CLPH-MA20.

Experiment	Time (min)	Hydrogel Mass (mg)	Dimethoate Retention (%)
1	10 (−1)	34.2 (−1.00024)	89.28
2	90 (+1)	34.3 (−0.99726)	100
3	10 (−1)	100.9 (0.98742)	100
4	90 (+1)	101.3 (0.99934)	100
5	50 (0)	65.1 (−0.07942)	100
6	50 (0)	60.1 (−0.22842)	100
7	50 (0)	65.6 (−0.06452)	100

Values between parentheses indicate the coded value used for the experimental design.

**Table 4:** List of dicarboxylic acids evaluated as crosslinking agents of different hydrogels.

Id.	Crosslinking Agent	Structure	Id.	Crosslinking Agent	Structure
1	Oxalic acid [31]	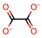	8	Itaconic acid [32]	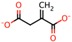
2	Malonic acid [31,32,33]	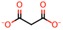	9	l-(+)-Tartaric acid [31]	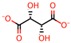
3	Succinic acid [31,32,34,35]	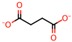	10	Glutaric acid [32,35,37,38]	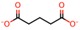
4	DL-Malic acid [36]	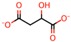	11	Adipic acid [31,32,35,37]	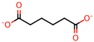
5	Fumaric acid [31,32]	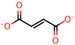	12	Pimelic acid [38]	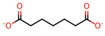
6	Maleic acid [31,32]	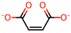	13	Suberic acid [38]	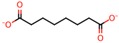
7	Citraconic acid [32]	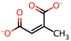	14	Azelaic acid [39]	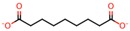

## Supplementary Materials

The following are available online at http://www.mdpi.com/2079-4991/8/1/23/s1, Figure S1: Methodology for design and study of intermolecular properties of hydrogels based on PVA polymers crosslinked with MA using molecular dynamic simulations. File S1: MOL2 file of the initial system CLPH-MA20/DMT/waters. File S2: MOL2 file of the initial system CLPH-MA40/DMT/waters. File S3: MOL2 file of the initial system CLPH-MA60/DMT/waters. File S4: CMS file of the initial system CLPH-MA20/DMT/waters.
