# Supplementary figures and images for: In-Silico Design, Synthesis and Evaluation of a Nanostructured Hydrogel as a Dimethoate Removal Agent
Source: Nanomaterials (Basel). 2018 Jan 4;8(1):23. doi: 10.3390/nano8010023 (PMC5791110; doi:10.3390/nano8010023)

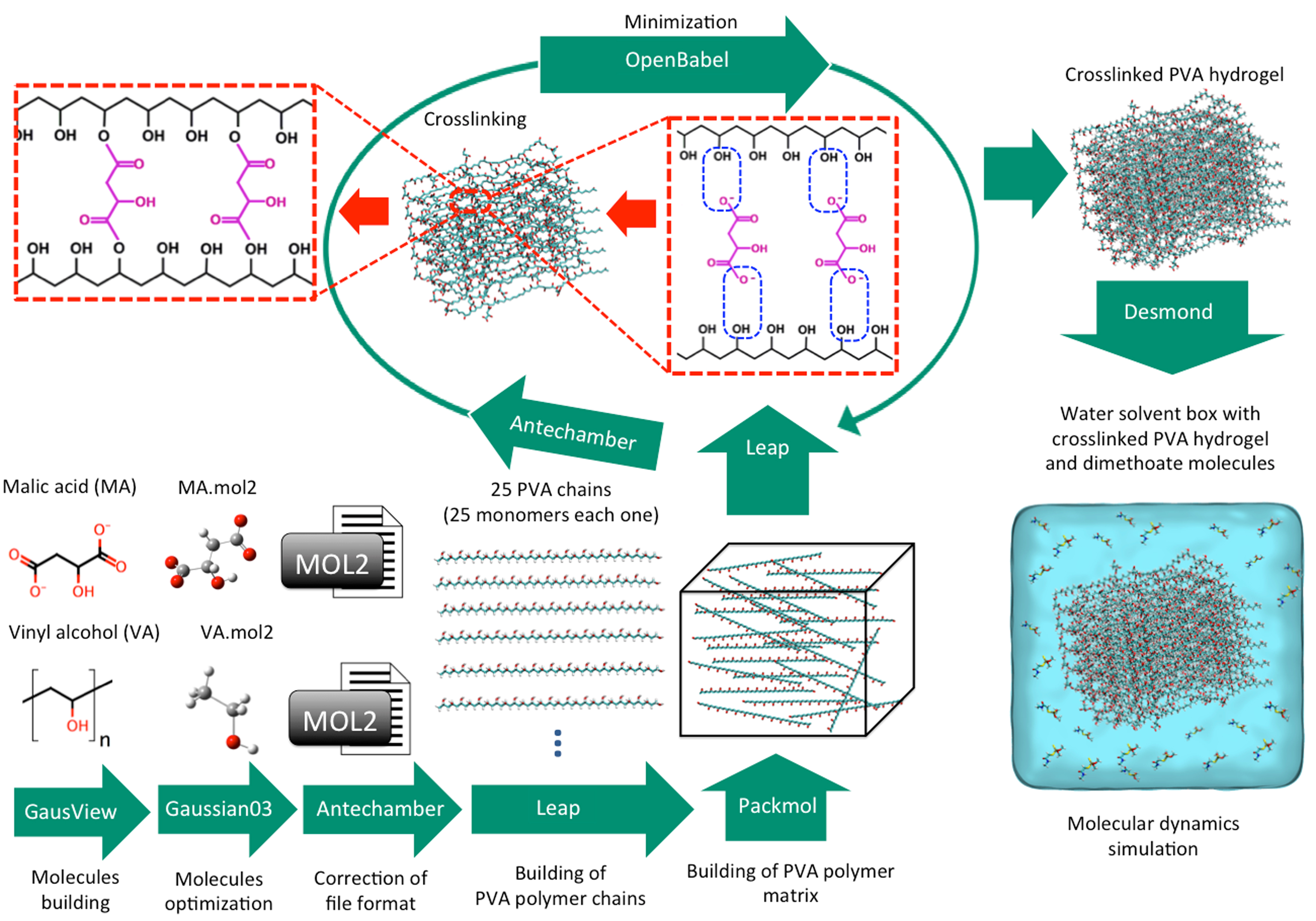

Supplement: Supplementary file 1 [file nanomaterials-08-00023-s001.zip › nanomaterials-253209-supplementary/supplementary-material/Figure S1.jpg]
